# Low-Grade Non-intestinal Type Sinonasal Adenocarcinoma: A Case Report

**DOI:** 10.7759/cureus.70774

**Published:** 2024-10-03

**Authors:** Abdulaziz F Alfadley, Abdullah Alhajlah, Naif AlOsaimi, Sarah S Al-Otaibi, Mohammad A Dababo, Ghassan Alokby

**Affiliations:** 1 College of Medicine, Alfaisal University, Riyadh, SAU; 2 College of Medicine, Imam Mohammad Ibn Saud Islamic University, Riyadh, SAU; 3 Otolaryngology and Head and Neck Surgery, Ministry of National Guard Health Affairs, Riyadh, SAU; 4 Otolaryngology and Head and Neck Surgery, King Faisal Specialist Hospital and Research Center, Riyadh, SAU; 5 Pathology and Laboratory Medicine, King Faisal Specialist Hospital and Research Center, Riyadh, SAU

**Keywords:** case report, mitotic figures, non-intestinal type adenocarcinoma, sinonasal adenocarcinoma, surgical management

## Abstract

Sinonasal non-intestinal-type adenocarcinomas represent a rare subset of head and neck cancers with distinct pathological and clinical characteristics. Patients usually present with nasal obstruction, epistaxis, and rhinorrhea. The diagnosis is established through histopathological examination. The treatment of choice is complete surgical excision with or without radiotherapy.

We report a case of low-grade non-intestinal type sinonasal adenocarcinoma in a 65-year-old male patient. Nasal endoscopy showed a left-sided grade 4 polyp based on Meltzer classification, which is attached to the left middle turbinate. Computed tomography of the paranasal sinuses showed a polyp in the superior part of the left nasal cavity compressing the left middle turbinate with obliteration of the left sphenoethmoidal recess. Initial biopsy results were inconclusive, and the patient underwent complete surgical excision of the mass along with anterior and posterior ethmoidectomy. Excisional biopsy results showed strong positivity for SOX10, S100, and DOG1, indicating seromucinous differentiation. Six months post-operatively, the patient is doing well with no clinical or radiological signs of recurrence.

In conclusion, this case underscores the importance of timely diagnosis and comprehensive surgical intervention in managing low-grade non-intestinal type sinonasal adenocarcinomas, ultimately contributing to improved patient outcomes and highlighting the need for further research in this rare cancer subtype.

## Introduction

Cancers of the nasal cavity and paranasal sinuses are infrequent, comprising less than 3% of upper respiratory tract cancers and under 1% of all cancer cases [1]. According to Turner and Reh [2], these neoplasms exhibit a notable male predilection with a male-to-female ratio of 1.8:1. Histologically, squamous cell carcinoma is the predominant subtype, accounting for 51.6% of cases, followed by adenocarcinoma, which accounts for 12.6%. The nasal cavity and maxillary sinus represent the primary anatomical locations for these neoplasms, with reported incidence rates of 43.9% and 35.9%, respectively [2].

According to the fifth WHO update on nasal, paranasal, and skull base tumors, primary adenocarcinomas originate from the respiratory surface epithelium or the subjacent seromucinous glands. These neoplasms are classified into two principal subtypes: salivary-type and non-salivary-type adenocarcinomas. The non-salivary-type, also known as surface epithelial adenocarcinomas, are subdivided into distinct entities known as intestinal-type adenocarcinoma (ITAC) and non-ITAC. Non-ITAC is further divided into high and low grades [3]. High-grade n-ITACs are rare types of cancers primarily found in the maxillary sinuses with a three-year survival rate of 30% [4]. In contrast, low-grade n-ITACs mainly affect the nasal cavity, ethmoid sinuses, and maxillary sinuses [5]. Histologically, they consist of uniform band cells forming papillary and glandular structures [5]. Low-grade n-ITACs have a good prognosis, with a five-year survival rate of 85% [6].

We herein report a rare case of low-grade non-intestinal type sinonasal adenocarcinoma in a 65-year-old male that was treated successfully.

## Case presentation

**Figure 1:**
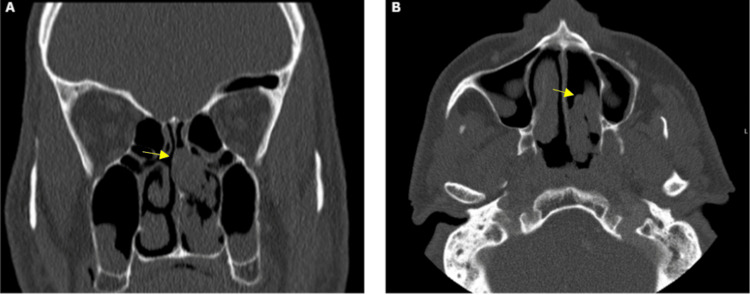
Computed tomography of the paranasal sinuses, coronal (A) and axial (B) views show a grade 4 polyp in the superior part of the left nasal cavity compressing the left middle turbinate and obliterating the left sphenoethmoidal recess as indicated by arrows

A 65-year-old heavy smoker male known to have ischemic heart disease, diabetes mellitus, hypertension, and nephrotic syndrome presented to our otolaryngology clinic with a one-year history of bilateral nasal obstruction and mucoid rhinorrhea, worse on the left side, associated with post-nasal drip and intermittent left-sided ear fullness. The patient denied facial pain or changes in his smell. Under endoscopic nasal examination, a left-sided grade 4 polyp based on Meltzer classification was seen attached to the middle turbinate, and the nasopharynx was partially seen [7]. For further evaluation, non-contrast computed tomography of the paranasal sinuses was performed (Figure 1). It showed a nasal polyp located in the inferior part of the left nasal cavity, abutting the left inferior turbinate and partially breaching the posterior choana with extension into the anterior aspect of the nasopharynx. Another nasal polyp located in the superior part of the left nasal cavity is compressing the left middle turbinate and obliterating the left sphenoethmoidal recess. An in-office biopsy was done under local anesthesia and was not optimally representative.

**Figure 2:**
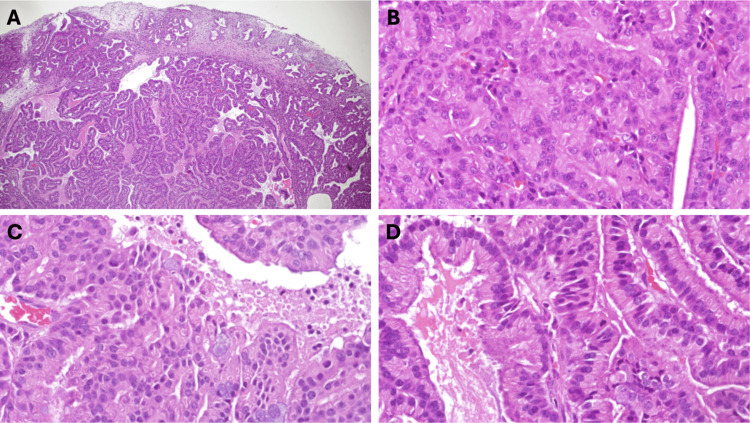
Hematoxylin and eosin show a polypoidal lesion with an unremarkable surface and subepithelial glandular proliferation (A). The glands are back-to-back with no intervening stroma in between. The cells are monomorphic with small nucleoli (B). The glands are mainly serous, with focal cells showing mucinous differentiation (C). High power magnification (x400) showing areas with papillary features and a mitotic figure as indicated by the black arrow (D)

The patient was booked for endoscopic sinus surgery, endoscopic resection of the left nasal mass, and endoscopic septoplasty. In surgery, a mass was seen on the left nasal cavity, which was attached to the medial surface of the middle turbinate. The middle turbinate was excised in the lower half along with the nasal mass. The remnant of the middle turbinate was cauterized. Then we proceeded with uncinectomy, ethmoidal bullectomy, maxillary antrostomy, and limited anterior and posterior ethmoidectomy followed by septoplasty. The patient tolerated the procedure well with no complications. During his first-week follow-up, the post-operative histopathology report was reviewed, and the diagnosis of “low-grade non-intestinal type sinonasal adenocarcinoma” was confirmed. Histological examination showed a polypoidal lesion with subepithelial serous glandular proliferation with focal cells showing mucinous differentiation. The glands are back-to-back without intervening stroma. The glands are lined by a single layer of cuboidal to columnar cells that are monomorphic with small nucleoli. Focal infiltration is noted along with scattered mitoses and a Ki67 proliferation index ranging from low (2%) to moderate (5%) with focal areas reaching up to 15% (Figure 2).

**Figure 3:**
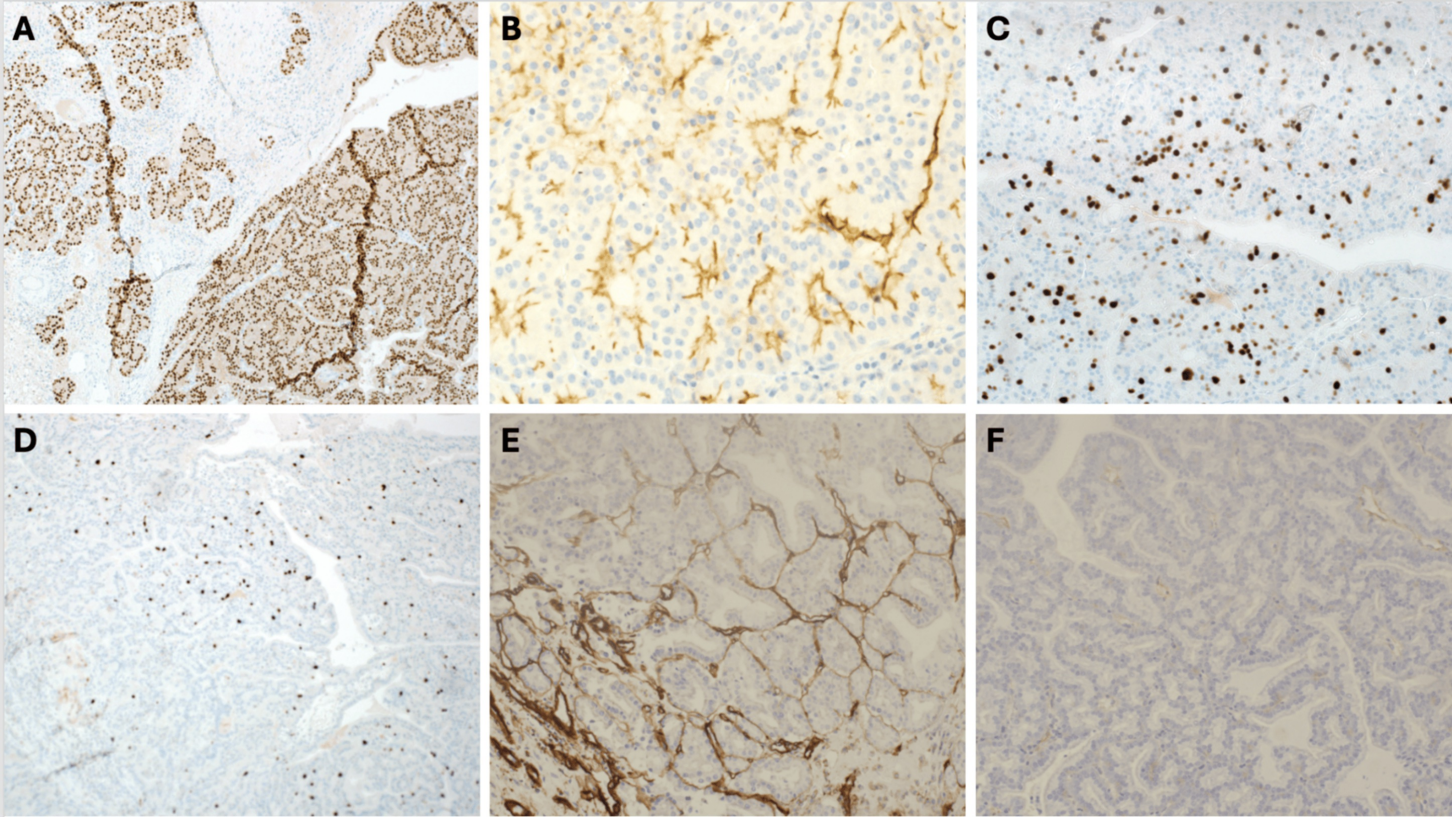
Immunohistochemical staining of the tumor shows strong positivity for SOX10 (A) and DOG1 (B). Ki-67 proliferation index ranges from low (2%) in the lower left to moderate (5%) in the upper right (C), with focally high areas reaching up to 15% (D). Focal areas of maintained staining noted in the original tiny biopsy show areas of collagen IV positivity between glands (E). Collagen IV complete loss of staining between glands (F)

Immunihestochemical staining revealed that the tumor cells were strongly positive for SOX10, S100, and DOG1 and negative for p63 (Figure 3). There is diffuse weak positivity for BRAF, for which molecular testing was negative. Margins could not be assessed due to specimen fragmentation; however, the turbinate tissue fragment shows a morphologically benign proliferation of seromucinous glands beneath the surface and does not appear to be involved by the tumor.

**Figure 4:**
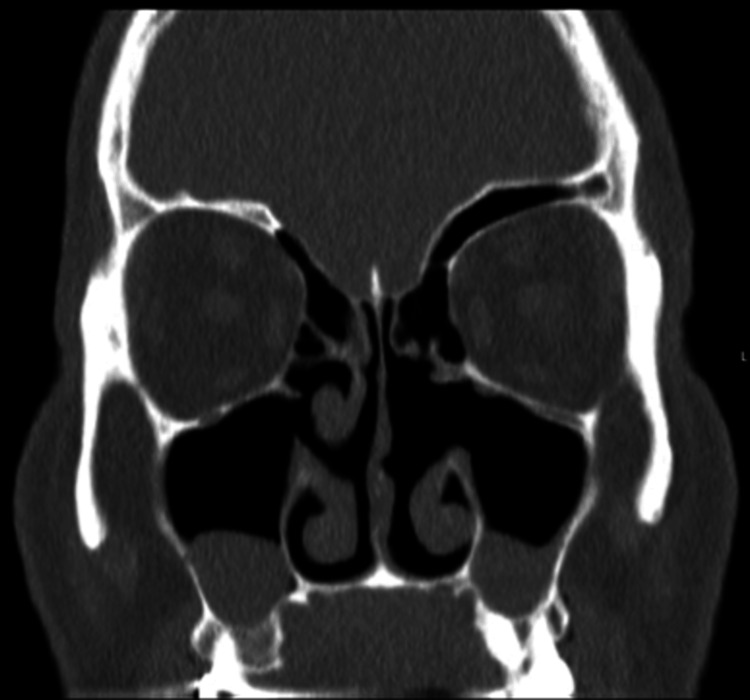
Post-operative computed tomography of the paranasal sinuses showing resolution of the primary disease

The staging workup was negative for metastasis. The patient’s case was discussed in the tumor board, and a decision was made for close follow-up with CT paranasal sinuses and a low threshold for repeat biopsy in the event of any changes in imaging and clinical examination. CT of paranasal sinuses six months post-operatively revealed mild soft tissue thickening involving left ethmoid and maxillary sinuses (Figure 4). The area was correlated with nasal endoscopic examination, and results were reassuring. Currently, the patient is asymptomatic and on regular follow-up in our clinic.

## Discussion

Non-intestinal-type adenocarcinomas (NITACs) are presumed to arise from seromucinous glands and are currently preferred to be called seromucinous carcinoma by some authorities [8]. These neoplasms are divided into high- and low-grade categories. High-grade NITAC are infrequent malignancies originating in the sinonasal tract. They predominantly affect males with a mean age of 60 years. Low-grade NITAC is a rare subset of sinonasal adenocarcinomas, comprising approximately 13% of cases. The ethmoid sinus is the predominant site involved, followed by the nasal cavity along the nasal turbinates and maxillary sinuses. The mean age of presentation ranges from 37 to 57 years [3,9]. Symptoms include nasal obstruction, epistaxis, rhinorrhea, and, in some cases, a visible tumor [10].

The histological architecture of low-grade NITAC is highly variable. Typically, the tumor displays an exophytic, papillary, or glandular growth pattern that is under 4 cm in the greatest diameter. The glands are lined by a single layer of cuboidal to columnar cells with the absence of a basal layer. However, the absence of a basal layer is not diagnostic of malignancy, as it is also seen in seromucinous hamartoma [8]. Pleomorphism and mitotic figures are infrequent. Back-to-back glandular proliferation with loss of collagen IV between glands and local invasion are indicative of malignancy. Immunohistochemistry shows consistent positivity for CK7 but is typically negative for CK20 and CDX-2. In addition, the absence of p63 indicates a lack of basal cells. A specific subset of low-grade NITAC has been designated as sinonasal seromucinous adenocarcinomas due to consistent expression of seromucinous differentiation markers, including DOG1, SOX10, and S-100 [9,11,12]. Our patient histopathology results showed a polypoidal lesion with subepithelial serous glandular proliferation with numerous back-to-back glands. Scattered mitosis and focal infiltration are noted. SOX10, DOG1, and S100 were all positive, indicating seromucinous differentiation.

The treatment of choice for non-ITAC patients is complete surgical excision. Endoscopic approaches are preferred and show lower morbidity, while radical approaches are rarely used. Regardless of the surgical approach employed, bilateral resection of the ethmoid sinus is crucial to reduce the risk of developing secondary primary tumors. This is due to histological evidence of the presence of tumor nests in unaffected mucosal regions distant from the primary tumor site. Post-operative radiotherapy may be necessary depending on the disease's extent and resection margins [4,9,11,13,14,15]. Yue et al. [16] conducted a retrospective analysis of 17 patients diagnosed with low-grade non-ITAC. All patients underwent complete surgical excision without radiotherapy. Of the 17 patients, only three experienced recurrence for which multiple surgical excisions were done, with no evidence of disease progression. The overall prognosis of low-grade non-ITAC is favorable, characterized by rare metastasis and deaths from the disease with a reported recurrence rate of 26% [14-16].

Our patient underwent a complete excision of the tumor along with anterior and posterior ethmoidectomy. The margins couldn’t be assessed in our case due to specimen fragmentation; however, the turbinate tissue fragment showed morphologically benign tissue. Six months post-operatively, CT of paranasal sinuses revealed mild soft tissue thickening. Clinical correlation with nasal endoscopy showed no findings, and the patient was reassured. Currently, the patient is asymptomatic and well.

## Conclusions

Sinonasal adenocarcinomas represent a rare subset of head and neck cancers with distinct pathological and clinical characteristics. This case highlights the challenges encountered in the diagnosis and management of a low-grade non-intestinal type sinonasal adenocarcinoma. Surgical excision remains the cornerstone of treatment, particularly in non-ITAC cases, with endoscopic approaches favored to minimize morbidity. Bilateral resection of the ethmoid sinus is essential to mitigate the risk of secondary primary tumors. Post-operative radiotherapy may be warranted based on disease extent and resection margins. Our patient's favorable outcome following complete tumor excision underscores the importance of meticulous surgical technique and close post-operative monitoring. Further research is needed to refine treatment strategies and optimize outcomes for patients with sinonasal adenocarcinomas.
